# Statistical inference for same data meta-analysis in neuroimaging multiverse analyzes

**DOI:** 10.1162/imag_a_00513

**Published:** 2025-03-31

**Authors:** Jeremy Lefort-Besnard, Thomas E. Nichols, Camille Maumet

**Affiliations:** Inria, Univ Rennes, CNRS, Inserm, IRISA UMR 6074, Empenn ERL U 1228, Rennes, France; Big Data Institute, Li Ka Shing Centre for Health Information and Discovery, Nuffield Department of Population Health, University of Oxford, Oxford, United Kingdom

**Keywords:** same data meta-analysis, multiverse analysis, reproducibility, task-fMRI, statistical inference

## Abstract

Researchers using task-functional magnetic resonance imaging (fMRI) data have access to a wide range of analysis tools to model brain activity. If not accounted for properly, this plethora of analytical approaches can lead to an inflated rate of false positives and contribute to the irreproducibility of neuroimaging findings. Multiverse analyses are a way to systematically explore pipeline variations on a given dataset. We focus on the setting where multiple statistic maps are produced as an output of a set of analyses originating from a single dataset. However, having multiple outputs for the same research question—corresponding to different analytical approaches—makes it especially challenging to draw conclusions and interpret the findings. Meta-analysis is a natural approach to extract consensus inferences from these maps, yet the traditional assumption of independence among input datasets does not hold here. In this work, we consider a suite of methods to conduct meta-analysis in the multiverse setting, which we call same data meta-analysis (SDMA), accounting for inter-pipeline dependence among the results. First, we assessed the validity of these methods in simulations. Then, we tested them on the multiverse outputs of two real-world multiverse analyses: “NARPS”, a multiverse study originating from the same dataset analyzed by 70 different teams, and “HCP Young Adult”, a more homogeneous multiverse analysis using 24 different pipelines analyzed by the same team. Our findings demonstrate the validity of our proposed SDMA models under inter-pipeline dependence, and provide an array of options, with different levels of relevance, for the analysis of multiverse outputs.

## 1 Introduction

The multiplicity of analytical methods, tools, and platforms available for modeling brain activity can have a substantial impact on neuroimaging findings (Botvinik-Nezer et al., 2020;Bowring et al., 2019;Glatard et al., 2015;Gronenschild et al., 2012;Strother et al., 2004). This flexibility in analysis combined with selective reporting may result in an increased occurrence of false positives and hence contributes to the lack of reproducibility in neuroimaging results. In departure to traditional analyses in which a single method is used, a multiverse analysis can be used to generate multiple outputs from the same dataset (Steegen et al., 2016). These various multiverse outputs arise from executing an array of pipelines, each representing a different framework for neuroimaging data analysis, which may include variations in both data processing and analysis steps. It is important to note that the multiverse analysis is not designed to distinguish between “correct” and “incorrect pipelines”, as all pipelines (used to generate the multiverse inputs) are assumed to be suitable to answer the research problem under study. Instead, the purpose of a multiverse analysis is to combine the results from these diverse pipelines to provide a more comprehensive exploration of the data, offering insights into how analytical variability can influence the results.

In this work, we develop methods for analytical pipelines that output a test statistic map for a particular effect of interest, that is, maps of T-scores or F-scores, both of which can be converted to Z-scores. We are motivated by task functional magnetic resonance imaging (fMRI) data, but our methods apply to any pipeline outputs producing test statistic images. While ideally we would work with parameter estimates and standard errors, across pipelines their units are often incompatible due to inconsistent scaling of the data, model and/or contrast, and thus we confine ourselves to Z-scores. The challenge is then how to combine these Z-scores to obtain valid and robust results.

Meta-analysis is a standard approach to combine the output of different studies. Current best practice is to combine parameter estimates and standard errors with a mixed-effects analysis with meaningful units (Normand, 1999); this approach accounts for between-study variance not explained by the standard errors of each study (Bossier et al., 2019). In neuroimaging, while these methods could, in principle, be applied for an Image Based Meta-Analysis (IBMA) however, this is challenging because researchers do not typically share effect size and standard error maps. Even if such maps were shared, each different software has a different approach to data and design matrix scaling that makes the units incompatible, and users can make arbitrary choices about contrast scaling as well. Thus practical IBMA is focused on combining test statistic images that have standardized units. The three most widely used methods for combining test statistics are Fisher’s, Tippet’s, and Stouffer’s, based on the sum of−logp-values, the minimum p-value, and the mean Z score, respectively (Lazar et al., 2002). However, all of these methods have been developed for independent inputs, not to combine outputs from different analytical approaches applied to the same dataset. In this work, we thus propose a set of dependence-adjusted meta-analysis methods—which we call “same-data meta-analysis” (SDMA)—accounting for inter-pipeline dependence among the multiverse outputs. We first assess the validity of the proposed SDMA methods on simulated multiverse outputs. Subsequently, we examine their relevance on the multiverse outputs of two distinct real-world multiverse analyses to gain deeper insights into the properties of each developed SDMA method. We conclude with a discussion of selecting the most suitable method based on specific use cases.

## 2 Methods

### 2.1 Models and estimators

Instead of Fisher’s and Tippet’s methods, we have elected to develop Stouffer’s method as it is the only combining method that is linear in the inputs. Thus, in the following we will develop five new same-data meta-analysis (SDMA) methods, three direct extensions of the Stouffer combining method, and two based on Generalized Least Squares for the optimal combination of dependent multiverse outputs.

#### 2.1.1 Input data

Broadly, there are three different types of outputs from a multiverse analysis: test statistics alone (e.g., only Z-score image), pairs of estimates and standard errors (e.g., as obtained from group task-fMRI analyses), and arbitrary values (e.g., correlations in connectome maps, or microstructural parameters from diffusion MRI). In this work, we consider only test statistics, leaving the other two cases for future work. Further, we assume all input maps take the form of Z-values (since other types of statistics can be converted to Z’s). Throughout, we further assume Normality, the basic assumption that would be required for statistical inference on any individual pipeline. Note that using z-values allows us to standardize the statistical maps, making them comparable across different pipelines and studies. This standardization ensures that the combined results are not biased by the varying scales of different estimators.

In the remainder of this manuscript, we adopt the following terminology:*‘dataset’*will refer to the original task fMRI dataset prior to any analysis;*‘multiverse outputs’*to the results of a multiverse analysis presented as a set of Z-maps (one for each pipeline); and*‘results’*to the statistical maps derived from applying a (same-data) meta-analysis model to the outputs of a multiverse analysis.

#### 2.1.2 Notation

We denote by$Y_{kj}$the value of the output for pipelinek=1,...,K, at voxelj=1,...,J. We assume these values are Z-scores, having mean zero and variance one under the null hypothesis, but allow for inter-pipeline correlation, aK×Kmatrix. We develop all of these methods assuming spatial homogeneity of correlation, that is, that all voxels share the same pipeline-to-pipeline correlationQ. This is a non-trivial assumption that we critically test on the multiverse outputs of real-world multiverse analyses (seeSection 2.2.4). Finally,Qconsistently refers to the inter-pipeline correlation; in this work focused on test statistics and significance testing validity, we can rely on a null hypothesis distribution ofN(0,1)for each input, and thus focus only on correlation.

#### 2.1.3 Conventional fixed-effects meta-analysis model: Stouffer method

The Stouffer method (Stouffer et al., 1949) is perhaps the most straighforward Z-score combining method, based on the sample mean of input Z-scores denoted${\bar{Y}}_{j}=\frac{1}{K}\sum_{k}Y_{kj}$:

$$Z_{j}^{\text{S}}=\frac{{\bar{Y}}_{j}}{\sqrt{1/K}}$$ (1)

where$Z_{j}^{\text{S}}$is again a Z score and has mean zero and variance one under the null and magnified mean$\mu_{j}\sqrt{K}$under the alternative where$\mu_{j}=E({\bar{Y}}_{j})$. This traditional meta-analytic is a fixed-effect method that is designed to powerfully combine evidence against the null. In essence, Stouffer combining creates an average map and then standardizes to account for$\text{Var}({\bar{Y}}_{j})=1/K$, producing a unit variance result.

#### 2.1.4 SDMA Stouffer

The standard Stouffer result is based on an assumption of independent inputs. Given that this assumption is not tenable in a multiverse setting, we propose a modification of the traditional Stouffer method, an SDMA version that accommodates an inter-pipeline correlationQ. First, note that the variance of an average ofKvariables with covarianceQis$1^{T}Q1\text{​}/\text{​}K^{2}$, where1is a vector of 1’s. We, thus, propose “SDMA Stouffer”$Z^{\text{SS}}$as the average with standardization to account for correlationQamong the inputs:

$$Z_{j}^{\text{SS}}=\frac{{\bar{Y}}_{j}}{\sqrt{{\text{1}}^{T}\text{Q}1/K^{2}}}.$$ (2)

#### 2.1.5 Consensus SDMA methods

While Stouffer methods scale the average to have variance 1.0 under the null, thus preserving the variance of each input Z-score, if the null is not true the scaling biases the estimate away from the mean of the inputs. With independent datasets this is natural—when multiple studies all have evidence against the null, their combined evidence is yet stronger evidence than the mean Z, as reflected by the$\sqrt{K}$-amplification in the magnified mean. With multiverse outputs, it is perhaps enigmatic: the original dataset is the same, but by combining similar but not identical versions of the multiverse outputs we can obtain results with amplified evidence against the null. Under the null hypothesis of mean zero signal everywhere, there is no concern of signal amplification, but when a signal is present it is impossible to scale a univariate (or single voxelj) average so that*both*mean and variance are preserved. However, for an image of statistics, we can shift the voxel-wise mean over voxels to have some target or “consensus” value.

In the following consensus methods, we propose two different ways to combineKtest statistic images such that the result is based on an average while preserving the voxel-wise mean and variance, yet the result is as similar as possible to theKmultiverse outputs fed into the method. In the following, we denote$\mu_{C}$and$\sigma_{C}$as the consensus mean and consensus standard deviation, respectively, we would like our final map to have. These could be set arbitrarily, but we assert that the most sensible values are the average over theKinputs

$$\mu_{\text{C}}=\frac{1}{K}\sum_{k}\langle {\text{Y}}_{k}\rangle$$ (3)

$$\sigma_{\text{C}}^{2}=\frac{1}{K}\sum_{k}\langle \langle {\text{Y}}_{k}\rangle \rangle$$ (4)

of the respective voxel-wise statistics, where〈⋅〉denotes image-wise average, that is,$\langle {\text{Y}}_{k}\rangle =\frac{1}{J}\sum_{j}Y_{kj}$is the voxel-wise average for inputk, and〈〈⋅〉〉is the voxel-wise variance, that is,$\langle \langle {\text{Y}}_{k}\rangle \rangle ={\left(J-1\right)}^{-1}\sum_{j}{\left(Y_{jk}-\langle {\text{Y}}_{k}\rangle \right)}^{2}$, and${\text{Y}}_{k}$is theJ-vector of Z-scores for pipelinek.

##### 2.1.5.1 Consensus SDMA Stouffer

Our first consensus method simply shifts the mean so that the image-wise mean of the output has the consensus mean:

$$Z_{j}^{\text{CSS}}=Z_{j}^{\text{SS}}-\langle Z^{\text{SS}}\rangle +\mu_{C},$$ (5)

where${\text{Z}}^{\text{SS}}$is theJ-vector image of SDMA Stouffer statistics. Note that this is just the SDMA Stouffer value centered image-wise to have average$\mu_{C}$. Of course, if the null hypothesis is true everywhere, then both$\langle {\text{Z}}^{\text{SS}}\rangle$and$\mu_{C}$will be zero with high precision (since they are an average over many voxels) and this will have no impact.

In summary, the Consensus SDMA Stouffer approach accounts for inter-pipeline correlation, but then adjusts the resulting map so that it has the voxel-wise average equal to the average overall all pipelines of the voxel-wise averages.

##### 2.1.5.2 Consensus average

The preceding SDMA Stouffer methods use the statistical theory to account for the impact of dependence on the variability of the computed summary. However, alternatively, a less technical approach is to simply compute an average and use its own voxel-wise statistics to standardize before scaling and shifting to have the desired consensus mean and standard deviation. Hence, we define the Consensus Average as

$$Z_{j}^{\text{CA}}=\frac{{\bar{Y}}_{j}-\langle \bar{Y}\rangle }{\sqrt{\langle \langle \bar{Y}\rangle \rangle }}\sigma_{C}+\mu_{C}$$ (6)

where$\bar{Y}$is theJ-vector voxel-wise average of theKinputs; note that here, we have set$\mu_{c}$equal to$\langle \bar{Y}\rangle$, but we maintain separate notation for$\mu_{c}$to accommodate the possibility of different choices in future work. While$Z_{j}^{\text{CSS}}$uses statistical results to compute the impact of averagingKdependent inputs,$Z_{j}^{\text{CA}}$simply uses the naive Stouffer as a starting point, standardizing, scaling, and shifting to desired consensus values. Though this approach makes slightly weaker assumptions by not assuming homogeneousQover space, it is expected that the Consensus Average$Z_{j}^{\text{CA}}$will produce values very similar to Consensus SDMA Stouffer$z_{j}^{\text{CSS}}$.

#### 2.1.6 SDMA GLS methods

##### 2.1.6.1 SDMA generalized least squares (GLS)

When analyzing dependent multiverse outputs, the optimal, minimum variance estimates are obtained by generalized least squares (GLS), where both data and model are whitened. First, consider the unwhitened case: For a regression of theK-vector of input data${\text{Y}}_{j}$on a design matrixX=1, the least squares estimate is${\left({\text{X}}^{⊤}\text{X}\right)}^{-1}{\text{X}}^{⊤}{\text{Y}}_{j}={\text{1}}^{⊤}{\text{Y}}_{j}/K$(the average) and the variance of the estimate is

$${\left({\text{X}}^{⊤}\text{X}\right)}^{-1}{\text{X}}^{⊤}\text{Var}({\text{Y}}_{j})\text{X}{\left({\text{X}}^{⊤}\text{X}\right)}^{-1}=1^{⊤}\text{Q}1/K^{2},$$ (7)

exactly the variance found above, and the estimate divided by standard deviation is exactly the SDMA Stouffer (2).

So now instead consider whitening with${\text{Q}}^{-1/2}$, giving GLS mean estimate

$${\bar{Y}}_{j}^{\text{G}}={\left(X^{⊤}Q^{-1}X\right)}^{-1}X^{⊤}Q^{-1}Y_{j}=\frac{1^{⊤}Q^{-1}Y_{j}}{1^{⊤}Q^{-1}1}$$ (8)

and variance

$${\left({\text{X}}^{⊤}{\text{Q}}^{-1}\text{X}\right)}^{-1}{\text{X}}^{⊤}{\text{Q}}^{-1}\text{ Var}\left({\text{Y}}_{j}\right){\text{Q}}^{-1}\text{X}{\left({\text{X}}^{⊤}{\text{Q}}^{-1}\text{X}\right)}^{-1}={\left(1^{⊤}{\text{Q}}^{-1}1\right)}^{-1}.$$ (9)

Thus, our SDMA GLS is the GLS estimate divided by its standard deviation:

$$Z_{j}^{\text{SG}}=\frac{{\bar{Y}}_{j}^{\text{G}}}{\sqrt{{\left({\text{1}}^{⊤}{\text{Q}}^{-1}\text{1}\right)}^{-1}}}=\frac{{\text{1}}^{⊤}{\text{Q}}^{-1}{\text{Y}}_{j}}{\sqrt{{\text{1}}^{⊤}{\text{Q}}^{-1}\text{1}}}$$ (10)

The motivation behind using GLS is that, instead of weighting each output equally as in$Z_{j}^{\text{SS}}$, we combine theKmultiverse outputs according to$1^{T}{\text{Q}}^{-1}$, which has the effect of down-weighting the influence of highly dependent pipelines. To illustrate, consider a scenario where the first half of multiverse outputs are derived from the same pipeline computed across various operating systems, resulting in virtually identical multiverse outputs. Conversely, the second half of pipelines produce nearly independent multiverse outputs. When calculating an unweighted average, equal weight is assigned to all inputs, while$1^{T}{\text{Q}}^{-1}$will give the first half of pipelines much less weight, approaching the influence of one individual independent pipeline.

##### 2.1.6.2 Consensus SDMA GLS

We can likewise define a Consensus SDMA GLS,$Z^{\text{CSG}}$, which is shifted to have a consensus image-wise average:

$$Z_{j}^{\text{CSG}}=Z_{j}^{\text{SG}}-\langle {\text{Z}}^{\text{SG}}\rangle +\mu_{C},$$ (11)

where${\text{Z}}^{\text{SG}}$is theJ-vector of SDMA GLS statistic values.

### 2.2 Evaluations

#### 2.2.1 Simulated multiverse outputs

##### 2.2.1.1 Null multiverse outputs generation

We simulated a set of Z-statistic maps under the null hypothesis according to aK-dimensional normal; at each voxelj, we have

$${\text{Y}}_{j}∼N\left(0,\text{Q}\right)$$

Note that by construction$Y_{j}$has variance 1.

In a first null scenario, “independent pipelines”, we generated multiverse outputs withμ=0andQ=I. In other words, there was no correlation between pipelines.

In a second null scenario, “correlated pipelines”, we setμ=0and considered different levels of dependence, specifically using compound symmetric correlation structures where all correlations are equal. The correlation was set to one of three possible values (0.2, 0.5, and 0.8). Specifically, all pipelines were correlated to the same degree, with the correlation level varying between 0.2, 0.5, and 0.8 depending on the simulation.

In a third null scenario, three pipelines were independent and the others were correlated pipelines, considering the same three possible values as above. Here, three pipelines were independent while the rest were correlated to the same degree, with the correlation level varying between 0.2, 0.5, and 0.8 depending on the simulation.

For each scenario, the number of pipelines and voxels were respectively varied,K∈{20;50;100} andJ∈{5,000;10,000;20,000}. This setup resulted in a total of 27 Monte Carlo realizations per scenario.

Note that all simulations provide a baseline scenario where no true effects are present, allowing us to assess the performance of the methods under ideal, null conditions.

Simulations were implemented in Python (3.11.6). Summary heatmaps for each of the main scenario can be found inFigure 1. All scripts to run the experiments and to create the figures and tables of this paper are accessible online,https://github.com/Inria-Empenn/SDMAand in Software Heritage public archive (“Software Heritage Identifier”, 2024).

**Fig. 1.**
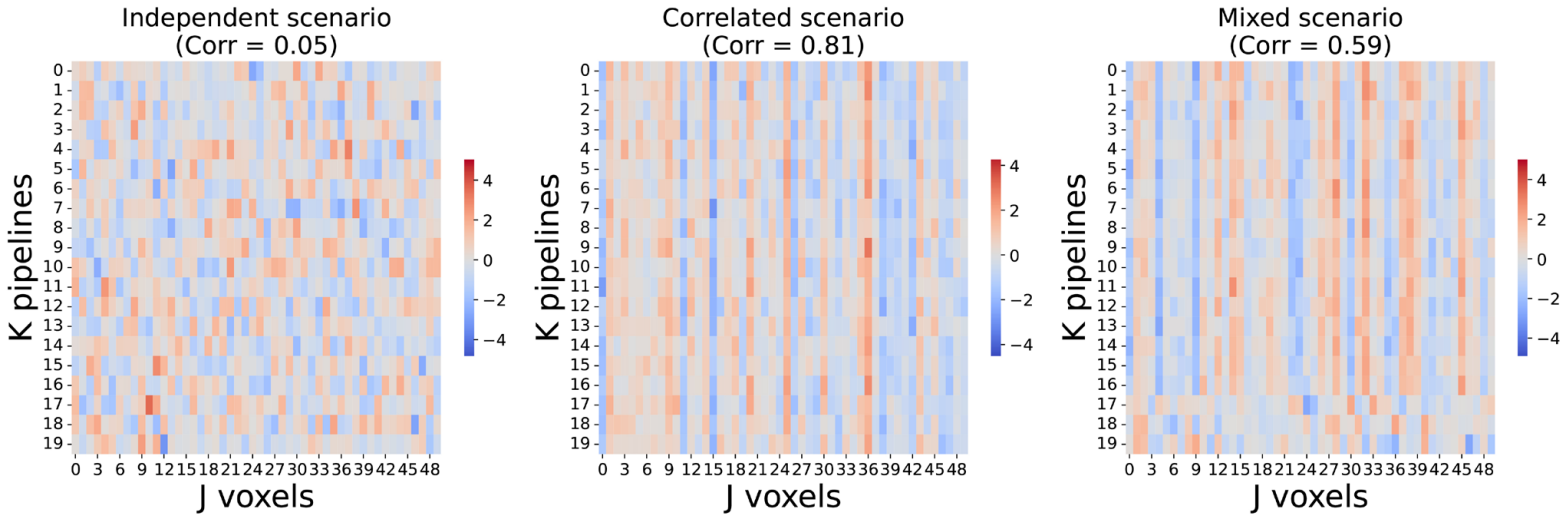
Illustration of the three different simulation scenarios (under the null hypothesis) using 1D images, each row corresponding to one 50-voxel image. The first setting has independent voxels and pipelines (left); in the second setting, there is dependence across pipelines (middle); and in the third mixed setting (right), 3 pipelines are independent (with a correlation of 0), while others are dependent (with a correlation of 0.8), giving an overall correlation of 0.59 as shown on top of the matrix.

#### 2.2.2 Assessment of validity

We evaluated the false-positive rate for each meta-analytic estimator using simulated null data. Each SDMA method generated Z-scores which were converted to p-values. These p-values were left uncorrected for multiple comparisons to enable a direct comparison between the SDMA methods. An SDMA method was deemed to perform well if the proportion of significant p-values less thanα=0.05was within the nominal 95% confidence interval; forJp-values this is$0.05\pm 1.96\sqrt{0.05\times 0.95/J}$.

To assess the validity of our method at levels other than 5%, we create comparative PP plots. While a conventional PP plot shows the ordered p-values$P_{\left(j\right)}$versus their expected value under the nullj/​(J+1), often in−logscale, divergence must be measured from the identity. To instead visualize departures from the horizontal, we plot the difference$-{\text{log}}_{10}P_{\left(j\right)}-(-{\text{log}}_{10}\left(j\text{​}/\text{​}\left(J+1\right)\right)$versus the reference value,$-{\text{log}}_{10}\left(j\text{​}/\text{​}\left(J+1\right)\right)$. This comparison is further aided by showing the 95% confidence intervals for uniform order statistics, based on the Beta distribution,B(j,J−j+1),$-{\text{log}}_{10}$transformed, and shifted by reference value$-{\text{log}}_{10}\left(j\text{​}/\text{​}\left(J+1\right)\right)$.

#### 2.2.3 Real-data multiverse analysis outputs

In the following sections, we will present two real-world datasets that have been analyzed using our multiverse approaches. It is crucial to keep in mind that, in multiverse analysis, all pipelines are assumed to be correct. This assumption allows us to explore the impact of analytical variability.

##### 2.2.3.1 NARPS multiverse outputs description

The Neuroimaging Analysis Replication and Prediction Study (NARPS) (Botvinik-Nezer et al., 2020) recently evaluated the degree and impact of analytic flexibility on task-fMRI results. They assessed the real-world variability of multiverse outputs across independent teams analyzing the same dataset. The dataset included task-fMRI data from 108 individuals, each performing one of two versions of a task previously used to study decision-making under risk (Canessa et al., 2013;Tom et al., 2007). This dataset is available on OpenNeuro (Markiewicz et al., 2021) at:https://openneuro.org/datasets/ds001734/versions/1.0.4. 70 teams were provided with the raw data and an optional preprocessed data, and were asked to analyze the data to test nine hypotheses, each consisting of a yes/no question regarding significant activity in a specific brain region in relation to a particular feature of the task. Among other outputs, each team submitted the unthresholded statistic maps supporting each hypothesis test. Access to these maps is described inhttps://github.com/poldrack/narps/tree/master/ImageAnalyses. In our study, we used the unthresholded statistic maps of 55 from the 70 teams included in NARPS. 15 statistic maps were excluded from the image-based analysis due to exclusion from the NARPS study or incomplete brain mask (seeSupplementary Table 1for details). We applied each of meta-analytic estimator on this set of 55 unthreshold maps, where all results were masked using a composite mask generated from the intersection of the MNI template and the pipelines’ brain masks, with a threshold of 0.9. This threshold was chosen because some pipelines produced relatively sparse results maps, and using a threshold of 1 would have excluded too many voxels. In this paper, we present our results within the first NARPS hypothesis. Results on the remaining hypotheses can be found in the Supplementary Material.

##### 2.2.3.2 HCP Young Adult multiverse outputs

The Human Connectome Project (HCP) Young Adult is an ambitious 5-year effort to characterize brain connectivity and function and their variability in healthy adults (Van Essen et al., 2012). It provides task-fMRI data for different tasks and cognitive processes. Using the motor task-fMRI data from the HCP Young Adult, the HCP multi pipeline dataset (Germani et al., 2023) provides multiverse outputs across 6 different contrasts and 24 different preprocessing and first-level analyses from the same dataset (for the 1,080 participants of the HCP Young Adults S1200 release). The 24 different pipelines differed in 4 parameters: software package (SPM or FSL), smoothing kernel (5 or 8 mm), number of motion regressors (0, 6, or 24) included in the General Linear Model (GLM) for the first-level analysis, and presence or absence of the derivatives of the Hemodynamic Response Function (HRF) in the GLM for the first-level analysis. Unthresholded statistic maps were obtained for each pipeline, resulting in 24 maps per contrast. In our work, we applied each of our meta-analytic estimators on the 24 multiverse outputs obtained for the right-hand contrast of the motor task. More details on the dataset can be found in the corresponding data paper (Germani et al., 2023). Note that these multiverse analysis outputs were generated within a single laboratory using only two software tools, in contrast to the NARPS multiverse outputs which involved 70 different teams and multiple software packages. As a consequence, the unthresholded maps of these HCP Young Adult multiverse outputs are more homogeneous than the 70 unthresholded maps of the NARPS mutliverse analysis, enabling us to examine the impact of heterogeneity on the proposed meta-analysis estimators.

#### 2.2.4 Assessment of Spatial Homogeneity of Correlation Q

Given that these SDMA methods assume that the inter-pipeline correlation is the same across the brain, we measured heterogeneity of this correlation within the multiverse outputs of the NARPS and HCP Young Adult analysis. We, thus, computed the magnitude of the difference between the inter-pipeline correlation when either using the whole brain or using subregions from a region-of-interest atlas.

First, we calculated the difference between correlation matrix of the whole brain and of a set of 7 brain regions (frontal, parietal, temporal, occipital, insular, cingulated, and cerebellum) derived from the AAL atlas (Tzourio-Mazoyer et al., 2002). This difference matrix highlights where and how much the matrices differ element-wise,

$${\text{Q}}_{\text{D}i}={\text{Q}}_{i}-{\text{Q}}_{b}$$

where${\text{Q}}_{i}$is the correlation matrix using one of the 5 brain regions and${\text{Q}}_{b}$the correlation matrix using the whole brain.

Then, the Frobenius norm of these difference matrices${\text{Q}}_{\text{D}i}$is computed, the square root of the sum of the squares of its elements, representing the magnitude of the difference between the two matrices,

$$∥{\text{Q}}_{\text{D}i}{∥}_{F}=\sqrt{\text{Tr}({\text{Q}}_{\text{D}i}^{⊤}{\text{Q}}_{\text{D}i})}$$

##### 2.2.4.1 Segmented analysis

Due to the relatively high Frobenius norm of the correlation difference observed in certain brain regions, we performed a subsequent segmented analysis allowing for a more detailed examination of spatial homogeneity.

Specifically, we applied each SDMA method separately for each brain region, and then assessed the discrepancies in significant activations for each brain region between the segmented and the whole-brain analysis using the Dice similarity index.

Writing significant voxels found using$Q_{i}$as setA, and significant voxels found by$Q_{b}$as setB, the Dice similarity indexDbetween is

$$D\left(A,B\right)=\frac{\left|A\cap B\right|}{2\times \left(\left|A\right|+\left|B\right|\right)}$$

where∩denotes set intersection and| · |cardinality.

#### 2.2.5 Interpretability of SDMA GLS results

As described below, we found a surprising level of divergence between GLS-based and the other methods. To help understand these differences, we developed an approach to measure the influence of each study on two types of methods, SDMA Stouffer and SDMA GLS.

Note that the SDMA Stouffer method (Eq. (2)) can be re-written

$$Z_{j}^{\text{SS}}=\sum_{k=1}^{K}w^{\text{Q}}Y_{kj}=w^{\text{Q}}\left(\sum_{k=1}^{K}Y_{kj}\right)$$ (12)

where

$$w^{\text{Q}}={\left({\text{1}}^{⊤}\text{Q1}\right)}^{-1/2},$$ (13)

showing that every studyk=1,...,Khas equal influence on the resulting statistic$Z_{j}^{\text{SS}}$.

Now consider rewriting SDMA GLS (Eq. (10)), as

$$Z^{\text{CGS}}=\sum_{k=1}^{K}w_{k}^{\text{QGC}}Y_{kj}$$ (14)

where

$$w_{k}^{\text{QGC}}={\left(1^{T}{\text{Q}}^{-1}1\right)}^{-1/2}\sum_{k^{\prime }}{\left(({\text{Q}}^{-1})\right)}_{k^{\prime }k},$$ (15)

which shows that each pipeline has an unequal contribution, determined according to the sum of each row of${\text{Q}}^{-1}$.

These expressions show that the weight of each pipeline is constant in SDMA Stouffer and equal to$w^{\text{Q}}$, while in SDMA GLS it varies as$w_{k}^{\text{QGC}}$.

To understand the behavior of GLS, we defined subgroups based on their similarities (see subgroup definitions below). Then, we calculated the SDMA Stouffer weights (Eqn. 13) as well as the SDMA GLS weights (Eqn. 15) assigned to each pipeline. We evaluated two key indicators:

1. **The contribution of each subgroup**, defined as the sum of contributions across all pipelines within the subgroup.
2. **The average weight**for each subgroup, which represents the mean of the weights assigned to each pipeline within the subgroup.

##### 2.2.5.1 Subgroups within the NARPS multiverse outputs

The authors of the NARPS study (Botvinik-Nezer et al., 2020) calculated Spearman correlations between whole-brain unthresholded statistic maps between each team and then clustered the pipelines based on similarities. The authors performed this clustering analysis for the nine hypotheses tested in NARPS. To assess and directly compare the performance of both the SDMA Stouffer and the SDMA GLS methods, we utilized their three subgroup solutions obtained within the first hypothesis, encompassing majority (highly correlated pipelines), opposite (anti-correlated pipelines), and unrelated (independent pipelines) subgroups (Supplementary Table 2). Given that SDMA GLS downweights the contribution of highly dependent pipelines, comparing the weight and contribution of various sets of pipelines might help visualizing SDMA GLS method behavior.

##### 2.2.5.2 Subgroups within the HCP Young Adult

Similarly to the approach taken in NARPS, we computed Spearman correlations among whole-brain unthresholded statistical maps from each of the 24 pipelines from (Germani et al., 2023), revealing highly correlated maps. Subsequently, we performed pipeline clustering based on these similarities and adopted the 2-cluster solution (Supplementary Fig. 8). We, thus, divided the 24 pipelines into 2 subgroups, namely FSL and SPM. Again, the weight and contribution of these two sets of pipelines were computed.

## 3 Results

### 3.1 Simulations results

**Fig. 2.**
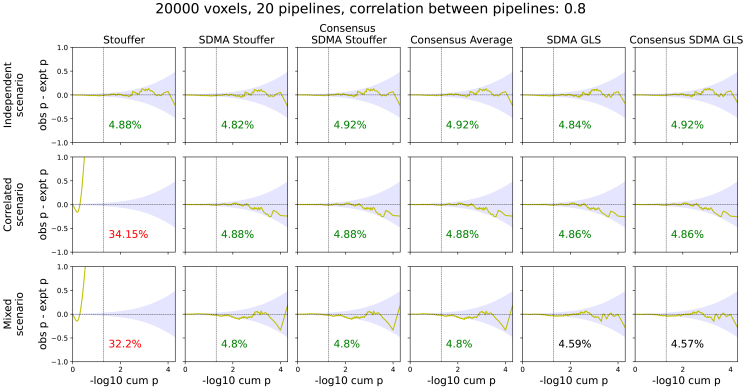
Comparative P-P plots for each meta-analysis estimator in the independent (upper row), correlated pipelines (middle row), and mixed (bottom row) simulations, where the y-axis is the difference in observed and expected -log10 ordered p-value, and the x-axis is the sorted expected -log10 ordered p-value. The blue shading depicts the nominal 95% confidence interval for each expected ordered p-value. At the bottom of each plot is the false-positive rate for nominalα=5%, displayed in red when significantly different from nominal, black when slightly outside the confidence intervals, and green otherwise. As expected, only the SDMA methods (all methods on the right of the “Stouffer”) performed well in the dependent multiverse setting.

Illustrative 1D simulated data are shown as images for each of the main scenarios inFigure 1. In simulations under the null scenario, where no effect was present, we find that when pipelines are independent (Fig. 2, upper row), all meta-analysis methods performed well (i.e., within the confidence bounds). However, in the correlated settings, we find that Stouffer method has a dramatically inflated false-positive rate whereas the SDMA estimators worked as expected (Fig. 2, middle row). The SDMA methods also control false positives when few independent pipelines were included in the correlated multiverse outputs (Fig. 2, bottom row). These results are shown for the 3 main simulations, withK=20pipelines,J=20,000voxels, and the correlation value is 0.8. Results were essentially identical for other combinations ofJ,K, and correlation values (Supplementary Figs. 1–4).

### 3.2 Real data: Homogeneity of **Q**

To assess the assumption that the correlationQis the same over the whole brain, we used normalized Frobenius norm on the difference betweenQcomputed over the whole brain versus individual brain regions. We found that these difference Frobenius norms were quite low for various brain regions in the HCP Young Adult multiverse outputs (Table 1), generally falling below 0.1. The only exception was white matter, which had a relatively higher score of 0.17, but should not be a concern for fMRI where results are only interpreted in gray matter regions. For the NARPS multiverse outputs, the difference Frobenius norm scores remained consistently low across four brain regions (frontal, parietal, temporal, and insular) in all NARPS hypotheses (Table 1), while they were relatively higher in eight other regions (occipital, cingulate, cerebellum, and white matter).

**Table 1.** Assessing the spatial homogeneity ofQin NARPS and HCP Young Adult multiverse outputs via the normalized Frobenius norm of the difference of regional vs. whole-brain computedQ.

	Difference Frobenius score							
	NARPS							
Brain region	Hyp 1	Hyp 2	Hyp 5	Hyp 6	Hyp 7	Hyp 8	Hyp 9	HCP
Frontal	0.07	0.07	0.10	0.09	0.10	0.08	0.07	0.07
Occipital	**0.18**	**0.13**	**0.13**	**0.20**	**0.14**	**0.20**	**0.12**	0.03
Parietal	**0.11**	0.09	0.08	0.08	0.09	0.08	0.09	0.06
Temporal	0.10	0.06	0.09	0.07	0.10	0.07	0.07	0.03
Insular	0.06	0.06	0.06	0.07	0.06	0.07	0.06	0.04
Cingulate	**0.11**	**0.11**	0.09	**0.14**	0.09	**0.14**	**0.11**	0.03
Cerebellum	**0.16**	**0.12**	**0.13**	0.09	**0.13**	0.09	**0.14**	0.03
White matter	**0.18**	**0.20**	**0.23**	**0.15**	**0.23**	**0.15**	**0.20**	**0.17**

While HCP has low values for all regions but white matter, most NARPS hypotheses have values above 0.1 for occipital, cingulate, and cerebellum in addition to white matter.

Values greater than 0.1 are in bold.

Given the relatively high Frobenius norm observed in some brain regions, we undertook a segmented analysis to more thoroughly investigate the impact of spatial heterogeneity. Specifically, for each SDMA method usingQ, we separately conducted the analysis within each region, and then assembled the results into a single image.

In the HCP multiverse outputs, we found a high level of overlap (Table 2) between the whole-brain results and individual brain regions results for methods based on the sample mean (SDMA Stouffer, Consensus SDMA Stouffer, and Consensus Average). Conversely, the Dice index showed a moderate level of overlap for methods involving whitening (SDMA GLS and Consensus SDMA GLS).

**Table 2.** Dice similarity of SDMA results maps for HCP data, comparing a global correlationQassumption and regionally specificQ, by region.

	Impact of regional vs. whole-brain Q for HCP results. Dice				
Brain region	SDMA Stouffer	Consensus SDMA Stouffer	Consensus Average	SDMA GLS	Consensus SDMA GLS
Frontal	0.97	0.96	0.96	0.75	0.75
Occipital	0.99	0.95	0.94	0.65	0.58
Parietal	0.99	0.99	0.99	0.96	0.96
Temporal	1.00	1.00	1.00	0.89	0.87
Insular	0.99	1.00	1.00	0.84	0.85
Cingulate	1.00	0.99	0.99	0.71	0.70
Cerebellum	1.00	0.98	0.98	0.67	0.62
White matter	0.87	0.89	0.89	0.59	0.58

Images were thresholded atα=0.05uncorrected. The methods based on the average (first three columns of results) all have high similarity, while GLS-based methods have poor similarity, reflecting the unstable influence of GLS’s whitening.

In the Narps multiverse outputs, the overlap (Table 3;Supplementary Tables 3–8) between whole-brain results and individual brain regions was more heterogeneous. For most NARPS hypotheses, methods based on the sample mean (SDMA Stouffer, Consensus SDMA Stouffer, and Consensus Average) demonstrated a high level of overlap while the Dice index indicated a moderate to low level of overlap for methods involving whitening. We observed a very high level of overlap for the SDMA Stouffer across all hypotheses and most brain regions, except for white matter, which consistently exhibited lower Dice values.

**Table 3.** Dice similarity of SDMA results maps for NARPS data, comparing a global correlationQassumption and regionally specificQ, by region. Images were thresholded atα=0.05uncorrected.

	Impact of regional vs. whole-brain Q for NARPS Hypothesis 1 results. Dice				
Brain region	SDMA Stouffer	Consensus SDMA Stouffer	Consensus Average	SDMA GLS	Consensus SDMA GLS
Frontal	0.98	0.6	0.61	0.88	0.83
Occipital	0.83	0.76	0.79	0.86	0.77
Parietal	0.97	0.96	0.99	0.85	0.85
Temporal	0.98	0.93	0.93	0.84	0.85
Insular	0.99	0.91	0.88	0.9	0.89
Cingulate	0.78	0.77	0.61	0.88	0.89
Cerebellum	0.8	0.82	0.8	0.93	0.95
White matter	0.78	0.98	0.88	0.81	0.47

SDMA Stouffer has the best similarity, indicating a relative robustness to the assumptions onQ, while still having reduced similarity on occiptal, cingulate, cerebellum, and white matter (consistent with difference Frobenius norm results inTable 1). Consensus methods show greater impact of regionalQ, and GLS methods even more so.

In summary, our findings indicate spatial homogeneity across gray matter, which is the area of primary interest. However, the heterogeneity observed in NARPS highlights the need for additional investigation to thoroughly understand and address this variability in a flexible and comprehensive way.

### 3.3 NARPS multiverse

**Fig. 3.**
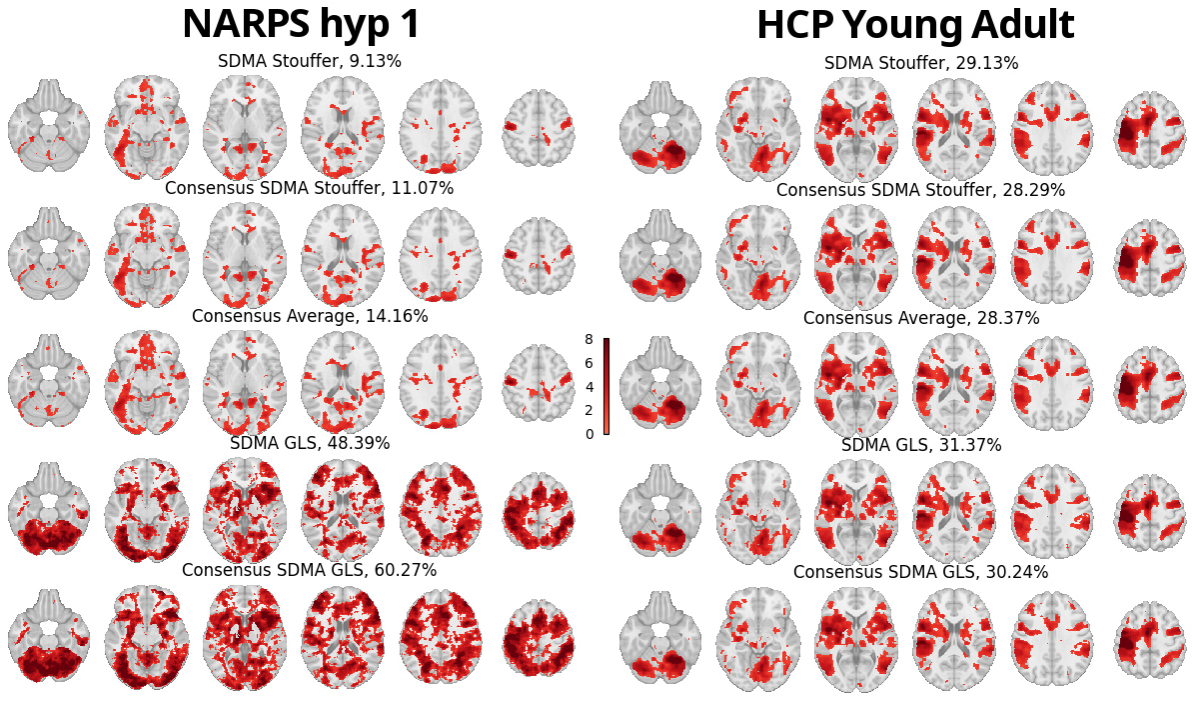
Relative comparison of meta-analysis estimators with significant Z-scores (p<0.05 uncorrected). Each row shows a different SDMA methods using the statistic maps from the NARPS study (first hypothesis, left panel) and using the statistic maps fromGermani et al. (2023)(*HCP Young Adult*, right panel). Name of the SDMA model and percentage of significant voxels are displayed on each map.

The meta-analysis estimators were calculated using the statistical maps from each of the 55 NARPS teams, producing Z-value and p-value maps. Significant Z-values (p<0.05 uncorrected) are displayed in MNI space.Figure 3(left) shows the results for the first NARPS hypothesis; seeSupplementary Figure 7for additional hypotheses. These maps are publicly available on NeuroVault (Gorgolewski et al., 2015) athttps://neurovault.org/collections/18197/.

Areas of significant activations were plotted inFigure 3(left column). The percentage of significant voxels within the analysis mask was similar in the SDMA Stouffer (9.13%), in the Consensus SDMA Stouffer (11.07%), and in the Consensus Average (14.16%). However, the GLS methods exhibited divergent outcomes, with a substantially higher proportion of significant voxels (48.39% and 60.27%). Note that unlike simulation scenarios, we are no longer operating under the null hypothesis and there is an unknown true signal. Proportions of voxels detected greater than 5% are expected and indicate the relative empirical power of each method.

### 3.4 HCP Young Adult multiverse

Combining the statistic maps from each of the 24 pipelines created a Z-value and p-value maps. Significant Z-values (p<0.05 uncorrected) are plotted in the same MNI space as NARPS (Fig. 3, right). These maps are publicly available on NeuroVault (Gorgolewski et al., 2015) athttps://neurovault.org/collections/18197/. In contrast to the findings of NARPS, all estimators yielded comparable results, ranging from 28.29% to 31.37% of significant voxels.

### 3.5 Comparison between SDMA Stouffer and SDMA GLS

**Fig. 4.**
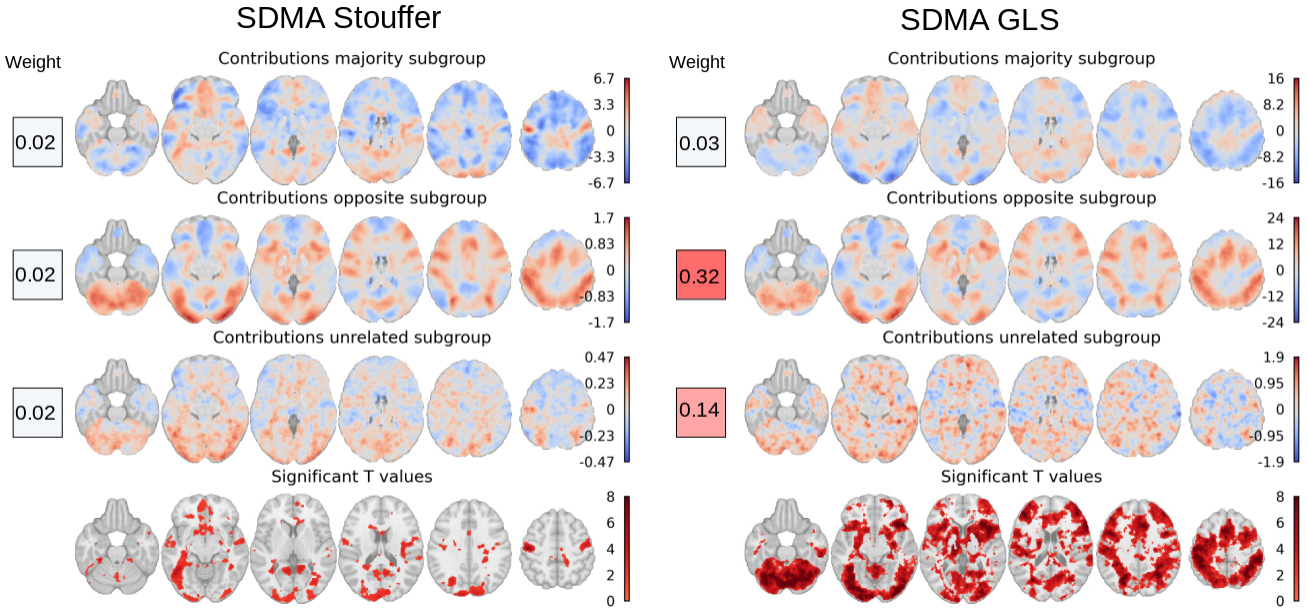
Characteristics of the SDMA Stouffer and the SDMA GLS methods illustrated on the NARPS multiverse outputs first hypothesis. The left panel illustrates the aggregated SDMA Stouffer contributions within each subgroup in MNI space, along with the mean SDMA Stouffer weight per subgroup (colored square). Likewise, the right panel showcased the aggregated contributions and average weights per subgroup, assigned by the SDMA GLS estimator. The bottom row displays the significance levels for each method. Our results indicate that SDMA Stouffer’s equal weighting benefits the majority group, whereas SDMA GLS emphasizes opposite subgroups. This is evident in the mean weights, which show that the majority group’s weight in SDMA GLS is reduced compared to that in the other subgroups.

Motivated by the differences observed in the results in the NARPS multiverse outputs, between equally weighted and whitened SDMA methods, we examined the weight and contribution assigned by SDMA Stouffer and SDMA GLS across three distinct pipeline subgroups in the NARPS multiverse outputs: majority, opposite (signed result), and unrelated subgroups. Our results showed that using the SDMA Stouffer method, the final significance map closely resembles the contribution map of the majority subgroup, which contains most of the pipelines (Fig. 4, left section). Equal weighting is allocated to every pipeline and consequently to each subgroup, resulting in the majority group exerting the greatest influence. Examination of weights and contributions per pipeline subgroup reveals that GLS attributed greater importance to the unrelated and opposite subgroups (Fig. 4, right section), with the majority of significant voxels originating from the opposite subgroup, a surprising result as the significant effects are, in fact, in the opposite direction of the largest collection of studies. The GLS method should in theory be optimal and, indeed, performs well with the HCP data and appears to have the greatest sensitivity. However, its unexpected results with the NARPS data suggest caution is warranted. Part of GLS whitening based the inversion ofK×Kcorrelation matrixQ. Noting that the inverse of a compound symmetric (all-equal) correlation matrix also has all-equal off-diagonals, ifQis approximately homogeneous we can expect the same of its inverse; conversely, when it is highly structured, even small perturbations can dramatically alter its inverse. This is perhaps the simplest explanation for the surprising and seemingly GLS results with the NARPS data.

## 4 Discussion

The primary objective of this paper is to introduce and assess several same-data meta-analysis (SDMA) techniques for combining test statistic images. As expected, we find that the traditional Stouffer method produces dramatically inflated false positive rates in the presence of correlation among pipelines. Conversely, we show that our SDMA Stouffer, Consensus SDMA Stouffer, and Consensus Average methods are valid, robust, and suitable for multiverse settings. However, while the SDMA GLS methods are valid in simulation, our findings with the NARPS multiverse outputs show that complex and negative dependencies among pipelines can lead to unexpected behavior.

### 4.1 The conventional Stouffer method fails to address the dependency structure within the multiverse outputs

The conventional Stouffer method is grounded in an assumption of independent inputs, and as expected we found greatly inflated false-positive rates in the presence of dependence. This inadequacy motivated the creation of the five different SDMA methods for combining multiverse outputs.

### 4.2 SDMA methods are valid in both independent and multiverse simulations

Every SDMA method developed in this work worked as expected in simulations of both independent and dependent multiverse outputs under the null scenario, producing nominal false-positive rates. The correlation degree among pipelines did not influence these findings, nor did the number of voxels and pipelines included in the analysis. Our simulation results indicate that the developed SDMA methods are suitable in the context of a multiverse setting.

### 4.3 Application of SDMA methods on homogeneous multiverse outputs

In our analysis using multiverse outputs of real-world multiverse analysis, all proposed SDMA methods produced nearly identical results when applied to homogeneous multiverse outputs. On the HCP Young Adult multiverse outputs—which are relatively homogeneous—we found that all five of our methods produced nearly identical results. Notably, the methods that should be theoretically optimal (using GLS whitening instead of equally weighted average) were the most sensitive, detecting more voxels than the other methods. Overall, these results indicate that the five developed methods are robust and consistent across scenarios with minimal variability. We also note that while the motivation for the Consensus SDMA Stouffer method was to reduce the magnification of the significance from combining distinct information across the different pipelines, there was not a substantial difference between Consensus and SDMA Stouffer (regarding the results obtained using HCP Young Adult multiverse outputs).

### 4.4 Application of SDMA methods on heterogeneous multiverse outputs

On the NARPS multiverse outputs—which exhibit appreciable heterogeneity with some teams exhibiting negligible or even negative correlation with the main subgroup—the SDMA Stouffer, the Consensus SDMA Stouffer, and the Consensus Average methods yielded virtually identical results but the GLS-based SDMA methods produced susbtantially different result maps. We investigated the source of these differences and found that they can be attributed to the presence of anticorrelated pipelines (opposite subgroup) in the NARPS multiverse outputs. Our examination of weights and contributions within each subgroup of pipelines indicates that GLS assigns more weight to the unrelated and opposite subgroups, while diminishing the impact of pipelines from the majority subgroup. In instances involving highly heterogeneous pipelines, interpreting the resulting outcomes can be difficult and could be unstable in the presence of anticorrelated or otherwise outlier pipelines. However, since our Q is estimated over the entire brain, we do not believe that the instability of weights is a significant issue; rather, it is a consequence of the complex pattern of dependence between the pipelines.

### 4.5 Interpipeline dependence may vary spatially

With the HCP data we found that results were largely the same whether we assumed global or region-specific interpipeline correlationQwhen using equally-weighted SDMA methods (Table 2, first 3 columns). The similarity was lower when using GLS-based methods (Table 2, last 2 columns). With NARPS we found that some regions did have slightly different results, and these were minimized for SDMA Stouffer (Table 3).

### 4.6 Overall recommendations

In theory, combining not-very-dependent inputs could result in SDMA Stouffer producing Z values larger than any input, which motivated our Consensus methods. In practice, we found that the Consensus results were quite similar to SDMA Stouffer and thus this ‘amplifying’ effect was not apparent in the two datasets we considered. Thus, among these five methods we recommend the SDMA Stouffer as the basic go-to method that is robust and easy to interpret. We also recommend using the SDMA Stouffer if there is suspicion that inter-pipeline dependence may vary spatially. If there is a concern that effects are being amplified, either Consensus SDMA Stouffer or Consensus Average can be used. Finally, if one has a relatively homogeneous set of multiverse outputs (i.e., the spatial variability in the z-maps across pipelines is reasonably low) and wants to maximize the statistical power, SDMA GLS should produce the optimal inference.

## 5 Conclusion and Future Work

Multiverse analyses offer a systematic approach to practically address analytical variability, an important driver of irreproducibility in neuroimaging research, by exploring and integrating variation across different analysis pipelines applied to the same dataset. In this study, our emphasis was on meta-analysis methods for combining statistic maps in the multiverse setting, which considers inter-pipeline dependence among multiverse outputs. Through simulations and assessments on two real-world multiverse analysis outputs, we verified the effectiveness of our proposed SDMA models. We found some evidence of heterogeneity of interpipeline correlation, motivating the need for methods that can adapt to spatial variation inQ. Furthermore, our findings underscored that GLS methods in scenarios with high heterogeneity may result in unclear and difficult-to-interpret outcomes, suggesting they may not be appropriate for application in a multi-expert context like NARPS. In summary, while the careful selection of pipelines remains the responsibility of practitioners, we recommend that multiverse analysis followed by a combination of the results should be a key methodological approach in neuroimaging research. Our results suggest that this approach would mitigate the risk of bias induced by different analytical pipelines and enhance the reliability of findings.

## Supplementary Material

Supplementary Material

## Data Availability

Access to the NARPS multiverse outputs is described inhttps://github.com/poldrack/narps/tree/master/ImageAnalyses. Access to the HCP Young Adult multiverse outputs can be found in the corresponding data paper (Germani et al., 2023). All scripts to run the experiments and to create the figures and tables of this paper are accessible online, athttps://github.com/Inria-Empenn/SDMAand in Software Heritage public archive (“Software Heritage Identifier”, 2024). The result maps of the SDMA estimators in NARPS and HCP are publicly available on NeuroVault (Gorgolewski et al., 2015) athttps://neurovault.org/collections/18197/.
