# Supplementary material for: Statistical inference for same data meta-analysis in neuroimaging multiverse analyzes
Source: Imaging Neurosci (Camb). 2025 Mar 31;3:imag_a_00513. doi: 10.1162/imag_a_00513 (PMC12319750; doi:10.1162/imag_a_00513)
Supplement: Supplementary Material [file imag_a_00513-supp.pdf]

Supplementary materials of *Statistical Inference for Same Data Meta-Analysis for Neuroimaging Multiverse Analyzes*

Jeremy Lefort-Besnard,[1] Thomas E. Nichols,[2*] Camille Maumet[1*]

[1]Inria, Univ Rennes, CNRS, Inserm, IRISA UMR 6074,

Empenn ERL U 1228, Rennes, France

[2]Big Data Institute, Li Ka Shing Centre for Health Information and Discovery,

Nuffield Department of Population Health, University of Oxford, Oxford, UK

[*]These authors contributed equally to this work

Corresponding Author: Camille Maumet camille.maumet@inria.fr

October 11, 2024

Contents

S1 Supplementary Tables **S3**

S1.1 Supplementary Table 1 . . . . . S3

S1.2 Supplementary Table 2 . . . . . S4

S1.3 Supplementary Table 3 . . . . . S5

S1.4 Supplementary Table 4 . . . . . S6

S1.5 Supplementary Table 5 . . . . . S7

S1.6 Supplementary Table 6 . . . . . S8

S1.7 Supplementary Table 7 . . . . . S9

S1.8 Supplementary Table 8 . . . . . S10

S2 Supplementary Figures **S11**

S2.1 Supplementary Figure 1 . . . . . S11

S2.2 Supplementary Figure 2 . . . . . S12

S2.3 Supplementary Figure 3 . . . . . S13

S2.4 Supplementary Figure 4 . . . . . S14

S2.5 Supplementary Figure 5 . . . . . S15

S2.6 Supplementary Figure 6 . . . . . S16

S2.7 Supplementary Figure 7 . . . . . S17

S2.8 Supplementary Figure 8 . . . . . S18

S1 Supplementary Tables

S1.1 Supplementary Table 1

NARPS Team name	Reasons of exclusion
1K0E	Excluded by the Narps study
L1A8	Excluded by the Narps study
VG39	Excluded by the Narps study
X1Z4	Excluded by the Narps study
16IN	Excluded by the Narps study
5G9K	Excluded by the Narps study
2T7P	Excluded by the Narps study
I07H	Incomplete brain mask
X19V	Artefact in masking
K9P0	Incomplete brain mask
XU70	Incomplete brain mask

Table S1: Teams Rejected from Narps Study

S1.2 Supplementary Table 2

Subgroup name	Included teams name
Majority	AO86, 43FJ, O21U, 3PQ2, 0JO0, I9D6, 51PW, 94GU, 0ED6, R5K7, SM54, B23O, O03M, DC61, X1Y5, UI76, 2T7P, 2T6S, 27SS, T54A, 1KB2, 08MQ, V55J, 3TR7, Q6O0, E3B6, L7J7, 9Q6R, U26C, 50GV, B5I6, R9K3, C88N, J7F9, 46CD, C22U, I52Y, E6R3, R7D1, 0C7Q, 6VV2, 98BT, 6FH5, 3C6G, L3V8, 0I4U, 0H5E, 9U7M
Opposite	80GC, 1P0Y, P5F3, IZ20, Q58J, 4TQ6, UK24
Unrelated	9T8E, R42Q, L9G5, O6R6, 4SZ2

Table S2: Teams included in each NARPS subgroup

S1.3 Supplementary Table 3

Brain region	DICE (NARPS Hypothesis 2)				
	SDMA Stouffer	Consensus SDMA Stouffer	Consensus Average	SDMA GLS	Consensus SDMA GLS
Frontal	0.99	0.94	0.93	0.86	0.8
Occipital	0.96	0.96	0.95	0.89	0.89
Parietal	0.97	0.97	0.96	0.9	0.9
Temporal	0.99	0.96	0.95	0.89	0.76
Insular	0.99	0.96	0.94	0.89	0.85
Cingulate	0.99	0.99	0.98	0.78	0.82
Cerebellum	0.93	1	0.99	0.85	0.71
White matter	0.84	0.75	0.78	0.37	0.65

Table S3: Like Table 3 in the main text, we find that SDMA Stouffer has the best similarity, indicating a relative robustness to the assumptions on $\mathbf{Q}$, while still having reduced similarity on cerebellum and white matter. GLS-based methods have poor similarity, reflecting the unstable influence of GLS's whitening

S1.4 Supplementary Table 4

Brain region	DICE (NARPS Hypothesis 5)				
	SDMA Stouffer	Consensus SDMA Stouffer	Consensus Average	SDMA GLS	Consensus SDMA GLS
Frontal	1	0.96	0.99	0.47	0.39
Occipital	0.97	0.98	0.98	0.41	0.58
Parietal	1	0.89	0.89	0.52	0.52
Temporal	0.98	0.76	0.79	0.6	0.02
Insular	0.99	0.99	0.99	0.65	0.8
Cingulate	1	0.87	0.85	0.59	0.04
Cerebellum	0.93	0.65	0.57	0.5	0.42
White matter	0.83	0.91	0.97	0.48	0.01

Table S4: Like Table 3 in the main text, we find that SDMA Stouffer has the best similarity, indicating a relative robustness to the assumptions on $\mathbf{Q}$, while still having reduced similarity on cerebellum and white matter. GLS-based methods have poor similarity, reflecting the unstable influence of GLS's whitening

S1.5 Supplementary Table 5

Brain region	DICE (NARPS Hyp 6)				
	SDMA Stouffer	Consensus SDMA Stouffer	Consensus Average	SDMA GLS	Consensus SDMA GLS
Frontal	0.93	0.87	0.87	0.64	0.80
Occipital	0.83	0.97	0.98	0.27	0.53
Parietal	0.89	0.69	0.63	0.76	0.48
Temporal	0.98	0.79	0.80	0.87	0.21
Insular	0.96	0.89	0.87	0.72	0.55
Cingulate	0.87	0.79	0.75	0.63	0.81
Cerebellum	0.98	0.88	0.90	0.30	0.65
White matter	0.69	0.63	0.72	0.66	0.72

Table S5: Like Table 3 in the main text, we find that SDMA Stouffer has the best similarity, indicating a relative robustness to the assumptions on $\mathbf{Q}$, while still having reduced similarity on occipital, parietal, cingulate, and white matter. GLS-based methods have poor similarity, reflecting the unstable influence of GLS's whitening

S1.6 Supplementary Table 6

Brain region	DICE (NARPS Hypothesis 7)				
	**SDMA Stouffer**	**Consensus SDMA Stouffer**	**Consensus Average**	**SDMA GLS**	**Consensus SDMA GLS**
Frontal	0.98	0.95	0.95	0.65	0.53
Occipital	0.97	0.96	0.95	0.67	0.40
Parietal	0.98	0.96	0.95	0.59	0.50
Temporal	0.98	0.86	0.84	0.64	0.16
Insular	1.00	1.00	0.99	0.73	0.66
Cingulate	0.99	0.95	0.96	0.48	0.13
Cerebellum	0.94	0.94	0.95	0.74	0.04
White matter	0.81	0.74	0.72	0.62	0.38

Table S6: Like Table 3 in the main text, we find that SDMA Stouffer has the best similarity, indicating a relative robustness to the assumptions on $\mathbf{Q}$, while still having reduced similarity on cerebellum and white matter. GLS-based methods have poor similarity, reflecting the unstable influence of GLS's whitening

S1.7 Supplementary Table 7

Brain region	DICE (NARPS Hypothesis 8)				
	SDMA Stouffer	Consensus SDMA Stouffer	Consensus Average	SDMA GLS	Consensus SDMA GLS
Frontal	0.94	0.99	0.99	0.63	0.41
Occipital	0.93	0.91	0.88	0.64	0.26
Parietal	0.94	0.97	0.99	0.59	0.67
Temporal	0.98	0.89	0.86	0.68	0.40
Insular	0.96	1.00	0.98	0.63	0.56
Cingulate	0.88	0.92	0.91	0.56	0.50
Cerebellum	1.00	0.91	0.89	0.69	0.66
White matter	0.69	0.75	0.89	0.64	0.48

Table S7: Like Table 3 in the main text, we find that SDMA Stouffer has the best similarity, indicating a relative robustness to the assumptions on $\mathbf{Q}$, while still having reduced similarity on cingulate and white matter. GLS-based methods have poor similarity, reflecting the unstable influence of GLS's whitening

S1.8 Supplementary Table 8

Brain region	DICE (NARPS Hypothesis 9)				
	SDMA Stouffer	Consensus SDMA Stouffer	Consensus Average	SDMA GLS	Consensus SDMA GLS
Frontal	0.95	0.84	0.83	0.50	0.05
Occipital	0.95	0.91	0.87	0.41	0.45
Parietal	0.86	0.93	0.92	0.57	0.21
Temporal	0.98	0.79	0.77	0.79	0.15
Insular	0.93	0.87	0.83	0.64	0.40
Cingulate	0.89	0.86	0.87	0.44	0.03
Cerebellum	0.83	0.67	0.63	0.62	0.08
White matter	0.69	0.96	0.91	0.58	0.00

Table S8: Like Table 3 in the main text, we find that SDMA Stouffer has the best similarity, indicating a relative robustness to the assumptions on $\mathbf{Q}$, while still having reduced similarity on cerebellum and white matter. GLS-based methods have poor similarity, reflecting the unstable influence of GLS's whitening

S2 Supplementary Figures

S2.1 Supplementary Figure 1

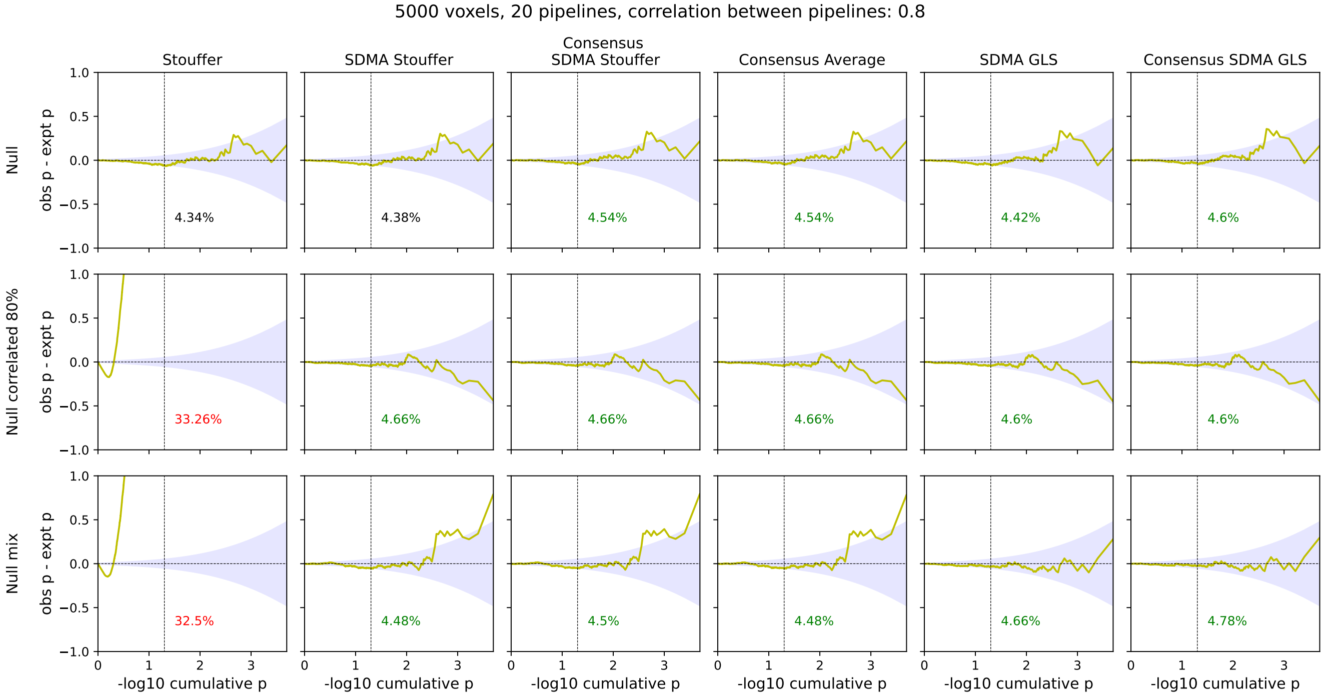

Supplementary Figure S1: Comparative P-P plots for each meta-analysis estimator in the independent (upper row), correlated pipelines (middle row), and mix (bottom row) simulations with 5000 voxels, 20 pipelines, and a correlation value of 0.8. The y-axis is the difference in observed and expected -log10 ordered P-value, and the x-axis is the sorted expected -log10 ordered P-value. The blue shading depicts the nominal 95% confidence interval for each expected ordered P-value. At the bottom of each plot is the false positive rate for $\alpha = 5\%$, displayed in red when significantly different from nominal, black when slightly outside the confidence intervals, and green otherwise.

S2.2 Supplementary Figure 2

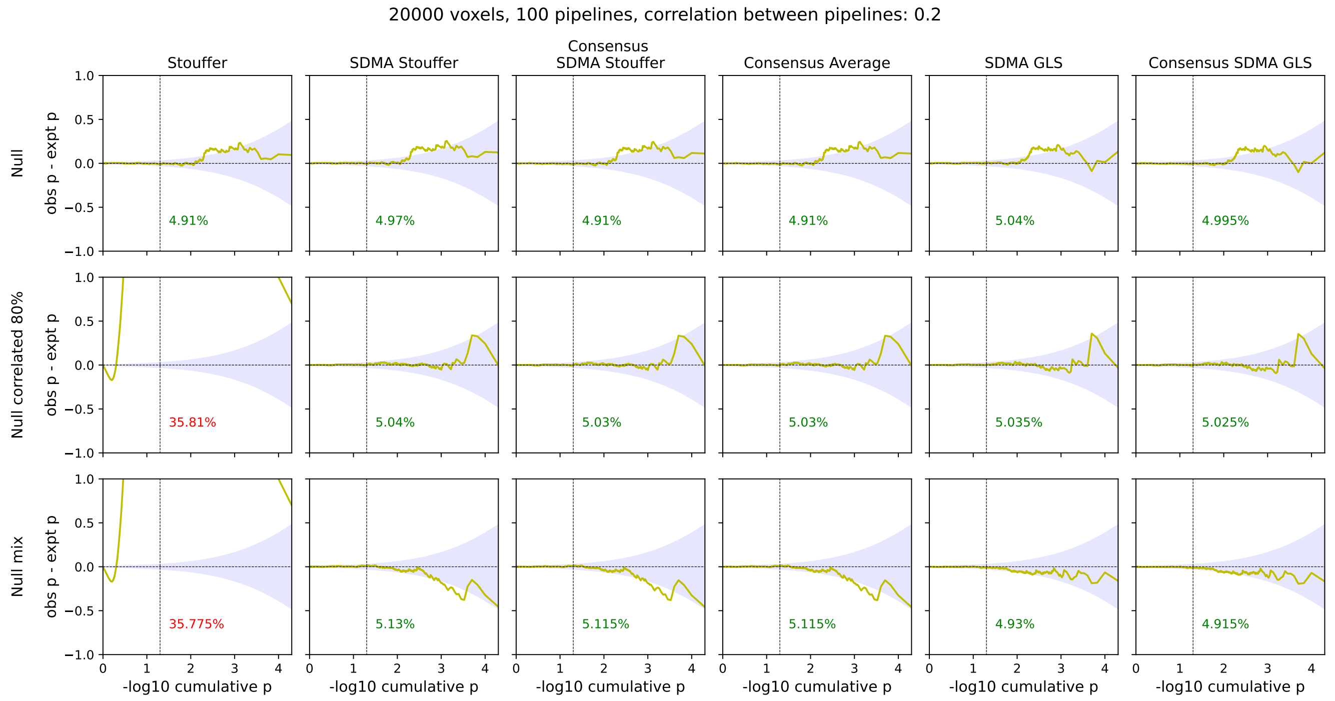

Supplementary Figure S2: See caption of Figure S1 for explanation of the plot.

S2.3 Supplementary Figure 3

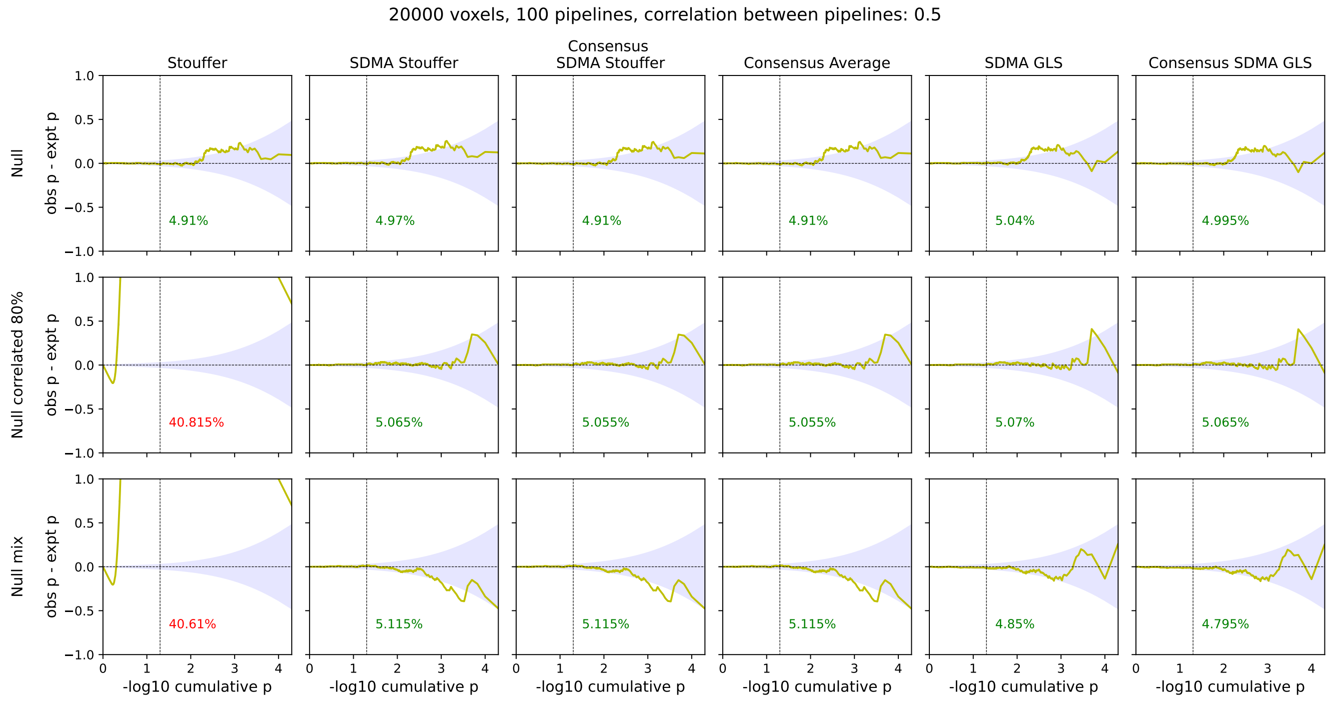

Supplementary Figure S3: See caption of Figure S1 for explanation of the plot.

S2.4 Supplementary Figure 4

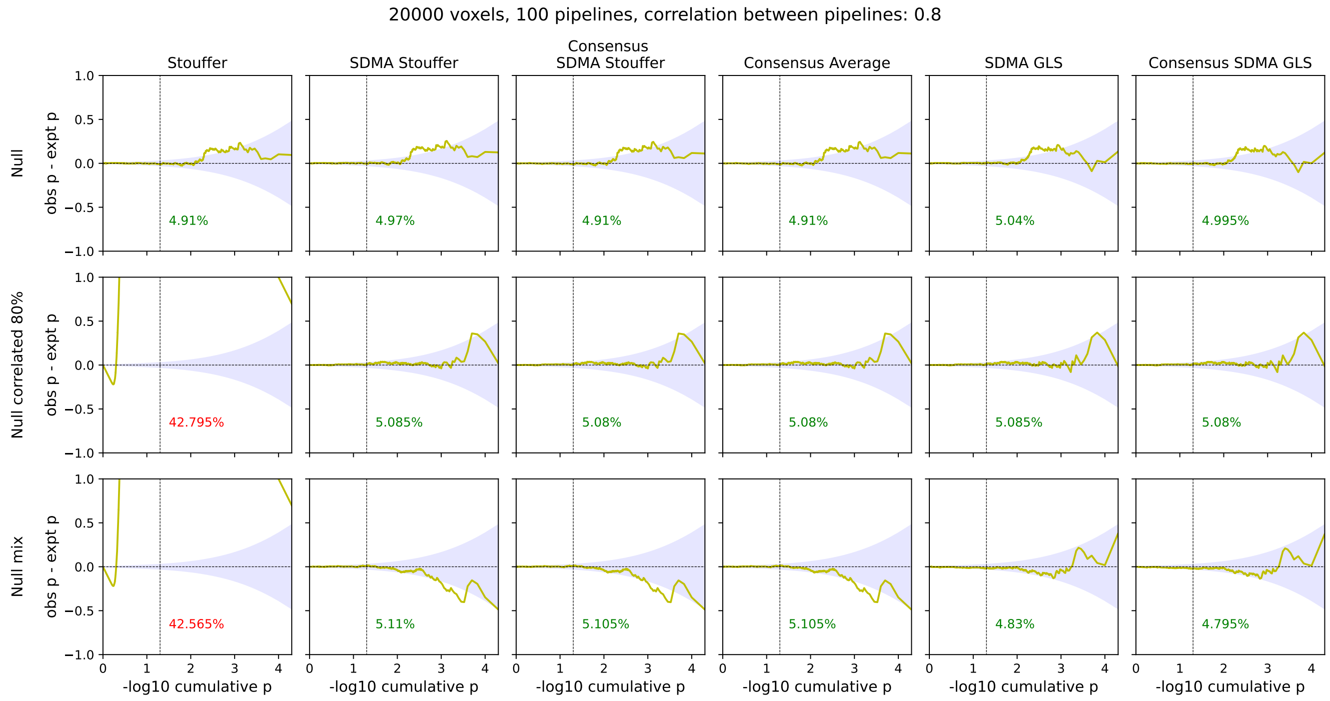

Supplementary Figure S4: See caption of Figure S1 for explanation of the plot.

S2.5 Supplementary Figure 5

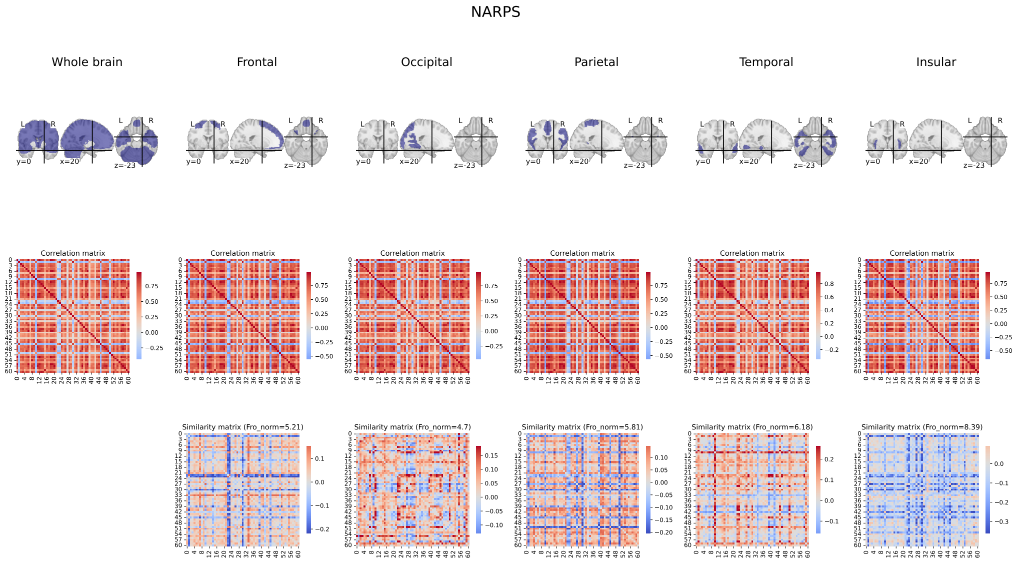

Supplementary Figure S5: The initial row presents the complete brain mask alongside the masks of a specific set of five brain regions. The subsequent row illustrates the correlation matrices calculated for both the entire brain and these five brain regions defined by the Harvard-Oxford Atlas (frontal, occipital, parietal, temporal, and insular). The subsequent rows exhibit the similarity matrices comparing the correlation matrix of the entire brain with each of the brain region correlation matrices using the NARPS team outputs, highlighting the specific discrepancies and their magnitudes on an element-wise basis. The Frobenius norm is indicated in the title of each similarity matrix.

S2.6 Supplementary Figure 6

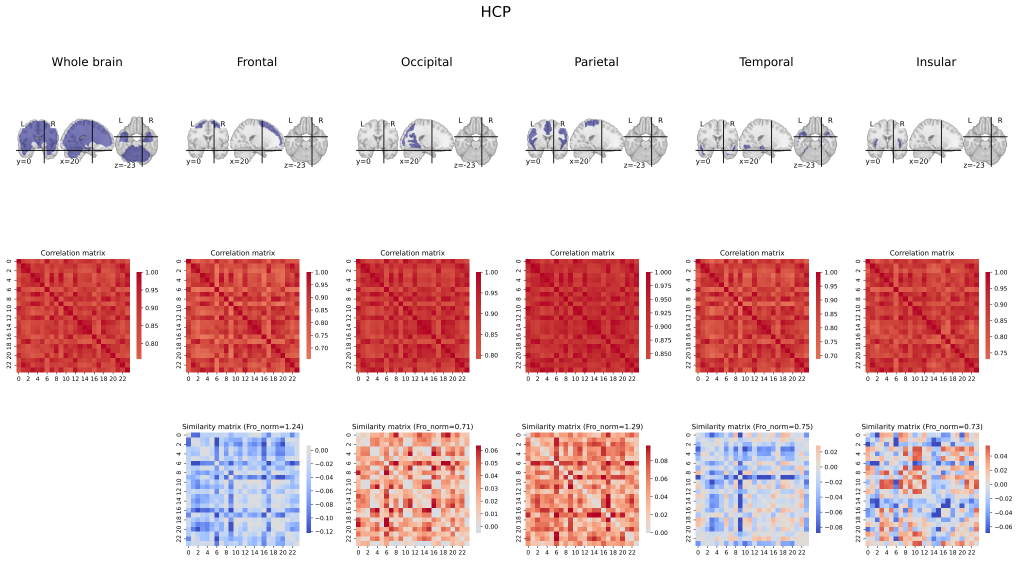

Supplementary Figure S6: See caption of Figure S5 for explanation of the plot, which displays the same information using HCP data instead of NARPS.

S2.7 Supplementary Figure 7

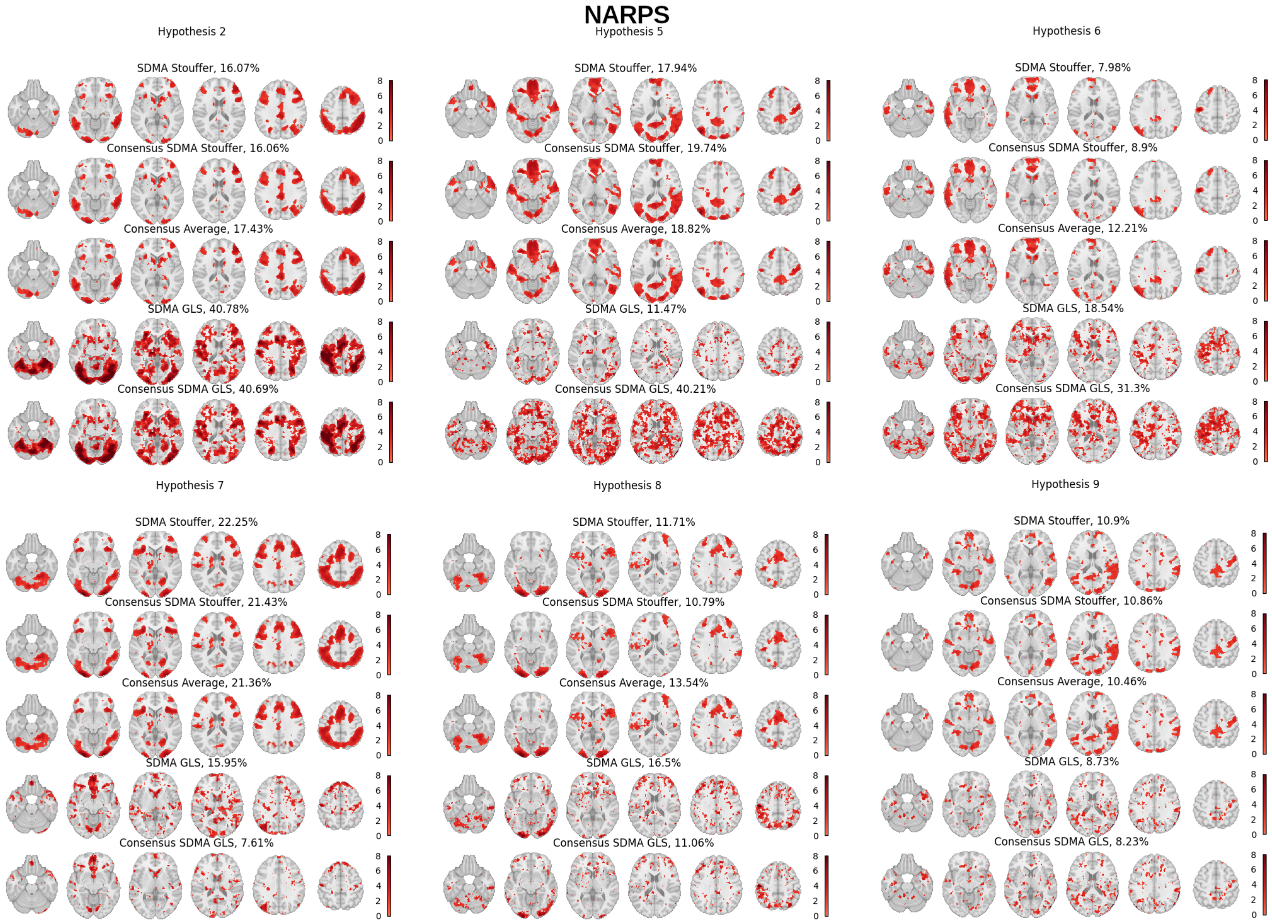

Supplementary Figure S7: Uncorrected significant P-values (indicated by their corresponding T-values) for each meta-analysis estimator and for each NARPS hypothesis. Demonstration of different SDMA methods using the statistic maps from the NARPS study for each hypothesis (1, 2, 5, 6, 7 8, and 9). Maps were thresholded at $p \leq 0.05$ uncorrected to allow for direct comparison. Name of the SDMA model and percentage of significant voxels are displayed on each map.

S2.8 Supplementary Figure 8

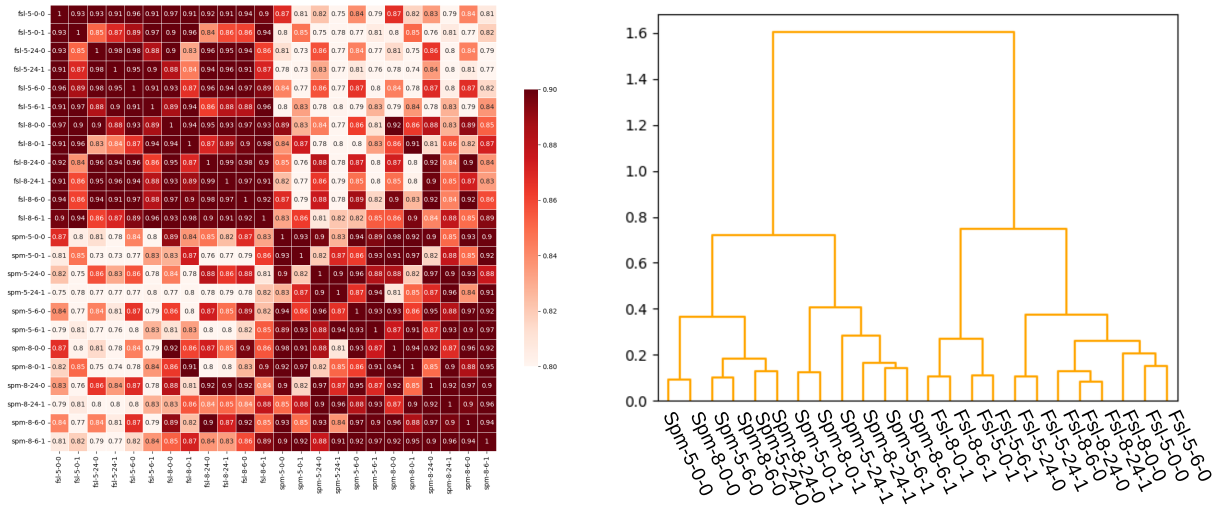

Supplementary Figure S8: The correlation matrix among HCP Young Adult pipelines is shown on the left. The clustering results based on these correlation scores are displayed on the right. Specifically, a 2-cluster solution was utilized for the analysis comparing SDMA Stouffer with SDMA GLS.
